# Temporal Dynamics of European Bat Lyssavirus Type 1 and Survival of *Myotis myotis* Bats in Natural Colonies

**DOI:** 10.1371/journal.pone.0000566

**Published:** 2007-06-27

**Authors:** Blanca Amengual, Hervé Bourhy, Marc López-Roig, Jordi Serra-Cobo

**Affiliations:** 1 UPRE Lyssavirus Dynamics and Host Adaptation, WHO Collaborating Center for Reference and Research on Rabies, Institut Pasteur, Paris, France; 2 Departament de Biologia Animal, Facultat de Biologia, Universitat de Barcelona, Barcelona, Spain; Université de Toulouse, France

## Abstract

Many emerging RNA viruses of public health concern have recently been detected in bats. However, the dynamics of these viruses in natural bat colonies is presently unknown. Consequently, prediction of the spread of these viruses and the establishment of appropriate control measures are hindered by a lack of information. To this aim, we collected epidemiological, virological and ecological data during a twelve-year longitudinal study in two colonies of insectivorous bats (*Myotis myotis*) located in Spain and infected by the most common bat lyssavirus found in Europe, the European bat lyssavirus subtype 1 (EBLV-1). This active survey demonstrates that cyclic lyssavirus infections occurred with periodic oscillations in the number of susceptible, immune and infected bats. Persistence of immunity for more than one year was detected in some individuals. These data were further used to feed models to analyze the temporal dynamics of EBLV-1 and the survival rate of bats. According to these models, the infection is characterized by a predicted low basic reproductive rate (R_0_ = 1.706) and a short infectious period (D = 5.1 days). In contrast to observations in most non-flying animals infected with rabies, the survival model shows no variation in mortality after EBLV-1 infection of *M. myotis*. These findings have considerable public health implications in terms of management of colonies where lyssavirus-positive bats have been recorded and confirm the potential risk of rabies transmission to humans. A greater understanding of the dynamics of lyssavirus in bat colonies also provides a model to study how bats contribute to the maintenance and transmission of other viruses of public health concern.

## Introduction

In the last ten years, many emerging RNA viruses have been detected in bat tissues [1]. These findings highlight the role of bats in the maintenance and transmission of viruses of public health concern. However, the way bats maintain and transmit these viruses is currently unknown. The same is true for *Lyssavirus*, viruses causing rabies in humans, for which bats are primary reservoirs on all inhabited continents [2]. Six of the seven *Lyssavirus* genotypes described to date infect bats. In Europe, two genotypes of *Lyssavirus*, European bat *Lyssavirus* types 1 and 2 (EBLV-1 and EBLV-2), circulate among several bat species [3]–[8,]. Numerous bats are found infected each year (http://www.who-rabies-bulletin.org) and the serotine bat (*Eptesicus serotinus*) appears to be the main victim of EBLV-1 infections in several European countries, according to data collected by passive surveillance [3], [7], [9]. These viruses can also cause a fatal illness, indistinguishable from classic rabies, in non-flying mammal species, including humans [3], [10]–[13]. Therefore, bat rabies is a public health concern in Europe. However, the epidemiology and the pathogenicity of EBLV in bats are still unknown. In particular, little data are available on the spatio-temporal dynamics of the infection and how this virus influences the mortality rate in bat colonies [5]. Studies addressing these issues require large databases, collected over the years, to monitor and assess local trends of rabies dynamics within a host population. These data are available for a limited number of mammal species [14]–[17] but are rare in bats [18], [8]. Misunderstanding about the role of bats as reservoirs and vectors of lyssaviruses have sometimes led to efforts to extirpate bat populations and to indiscriminately reduce these colonies, in spite of being protected species in many countries [92/43 and 97/62 EU Directives, 19,20].

Six European insectivorous bat species (*E. serotinus, Myotis myotis, Myotis nattererii, Miniopterus schreibersii, Rhinolophus ferrumequinum and Tadarida teniotis*) were shown to be infected in Spain by EBLV-1 through active survey [6], [18]. Here we have focused on *M. myotis*, which has a wide distribution throughout Europe, mainly in the south and center of the continent [21]. We provide the first report on the temporal dynamics of lyssavirus and survival bat in a natural colony based on a long term (12 years) longitudinal cross-sectional study of the prevalence of EBLV-1 neutralizing antibodies and EBLV-1 RNA in samples of that species. We also estimate, for the first time, the mortality rate in *M. myotis* before and after infection by EBLV-1 and describe the epidemiological characteristics of EBLV-1 infection in colonies of the same species by means of a simple temporal dynamic model, which is further validated by data.

## Results

### Ecology of *M. myotis*

Analysis of the cytochrome b gene demonstrated that the captured bats belonged to the species *M. myotis* (data not shown). Refuge 1 shelters a breeding colony of 212 (95% confidence interval [CI], 162–300) *M. myotis* (referred to herein as colony 1). They reach the cave in mid-April each year and begin to leave the cave at the beginning of August. Refuge 2 shelters a breeding colony of 591 (95% CI, 377-804) bats from spring to fall (referred to herein as colony 2). Recapture of banded bats showed only three exchanges of *M. myotis* between these 2 refuges, which are 35 km apart (Table S1).

### Fluctuation in the percentage of seropositive bats

**Figure 1:**
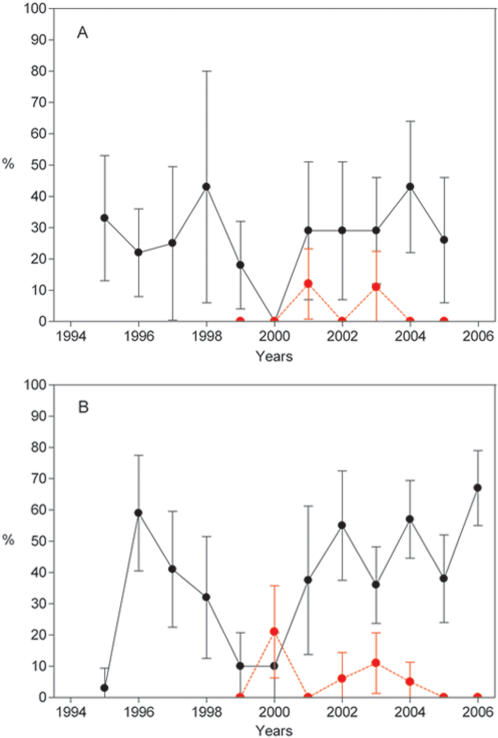
Temporal Variation of the Percentage of Sera and Blood Clot Positives in Bat colonies. Percentage of seropositive (black symbols) and blood clot positive (red symbols) bats observed in *M. myotis* colonies 1 (A) and 2 (B) (95% confidence interval shown).

Neutralizing antibodies revealed a high frequency of exposure (36.24%, n = 643) to EBLV-1 in *Myotis* bats, with some differences between colonies. The seroprevalence rate between adults and juveniles and between males and females was not significantly different in these colonies. No significant differences in the percentage of seropositive bats was observed in colony 1 (Chi-square χ ^2^ = 6.55, df = 9, p>0.5) during the study period (1995-2005) (Figure 1A). Therefore, this colony was considered at equilibrium and the average percentage of susceptible bats obtained during this period (58.62%, 95% CI, 51.99%–65.25%) was used to calculate the basic reproductive rate (R_0_ = 1.706, 95% CI, 1.533 – 1.923).

In contrast, significant inter-annual variations in the percentage of seropositive bats were observed in colony 2 (Chi-square χ ^2^ = 69.56, df = 9, p>0.000.1; Table 1). Four peaks in seropositive animals were identified from 1995 to 2006: 1995–1996 (χ ^2^ = 18.92, p<0.001); 2000–2002 (χ ^2^ = 12.57, p<0.001); 2003–2004 (χ ^2^ = 4.03, p<0.05); 2005–2006 (χ ^2^ = 7.33, p<0.001) (Figure 1B).

**Table 1:** Results from serologic and antigenic analyses in *M. myotis.*

Locality		1995	1996	1997	1998	1999	2000	2001	2002	2003	2004	2005	2006	TOTAL
Colony 1	A/B	7/21	7/32	3/12	3/7	5/28	0/6	5/17	5/17	8/28	9/21	5/19	nd	57/208
	X±SD	122±45	207±159	218±136	412±454	106±61	NA-	65±40	68±32	159±125	67±31	81±68	nd	
	Range	83–195	53–442	129–374	87–930	29–176	NA	56–126	36–117	36–334	36–107	35–190	nd	
	C/D	nd	nd	nd	nd	0/28	0/6	2/17	0/17	3/28	0/21	0/19	nd	5/136
	E/F	nd	nd	nd	0/2	0/4	nd	nd	nd	nd	nd	nd	nd	0/6
Colony 2	A/B	1/30	16/27	11/27	7/22	3/30	3/30	6/16	17/31	22/59	35/61	17/45	38/57	176/435
	X±SD	90	348±237	191±225	718±657	78±27	58±42	37±24	55±17	87±79	115±81	62±45	115±47	
	Range	NA	49–908	29–783	79–1677	47–95	29–107	31–94	39–123	36–348	36–370	29–146	35–177	
	C/D	nd	nd	nd	nd	0/30	6/29	0/16	2/32	4/38	3/43	0/45	0/57	15/290
	E/F	nd	0/2	nd	nd	0/4	1/3	0/1	nd	1/1	nd	nd	nd	2/11

A, number of seropositive bats. B, number of bat sera analyzed. X, mean seroneutralization titer of positive sera. SD, standard deviation. C, number of positive blood clots. D, number of blood clots analyzed. E, number of positive dead bats. F, number of dead bats analyzed. nd, not done, NA, non applicable.

The re-sampling of bats (2 to 4 times) at intervals between 1 and 8 years allowed us to follow the seroneutralization titer of 46 individuals over the years (Table 2). Seven individuals were captured and analyzed more than twice at intervals of one or several years. One bat was even analyzed four times between 1996 and 2004. This monitoring demonstrated that anti-EBLV-1 antibodies remained detectable in some *M. myotis* for at least one year after seroconversion. Nineteen of these 46 bats show positive antibody titers becoming negative in the following recapture sessions after various intervals in years. This indicates that these bats survive at least several years after their seroconversion.

**Table 2:** Individual Serological Follow-up in Recaptured Bats.

Locality	Year_1_	S_1_	C_1_	Year_2_	S_2_	C_2_	Year_3_	S_3_	C_3_	Year_4_	S_4_	C_4_
Colony 1	2002	45.8	-	2003	85.8	-						
	2002	0	-	2005	189.8	-						
	2003	65	-	2004	52.7	-	2005	0	-			
	2003	0	-	2004	35.5	-						
	2004	35.5	-	2005	126.6	-						
	2004	0	-	2005	35.5	-						
	2004	106.6	-	2005	0	-						
Colony 2	1996	0	nd	2002	62.6	-	2003	44.4	-	2004	0	-
	1996	709	nd	2002	69.2	-						
	1997	0	nd	2001	35.5	-						
	1997	29.3	nd	2001	0	-						
	1997	783.2	nd	2001	35.5	-						
	1997	0	nd	2004	67	+						
	1998	79.5	nd	2003	0	-	2004	0	-			
	2000	29	-	2001	38.8	-						
	2000	0	-	2001	40.5	-						
	2000	0	-	2002	50.3	-						
	2000	39	-	2002	48.6	-	2003	38.7	-			
	2001	35.5	-	2003	105.3	-						
	2002	84.1	-	2003	0	-						
	2002	62.6	-	2003	71.1	-						
	2002	52.7	-	2003	0	-						
	2002	110.3	-	2003	349	-						
	2002	0	-	2004	85.8	-						
	2002	94.8	+	2004	35.5	-						
	2002	116.2	-	2004	107	-	2006	169	-			
	2002	81.4	-	2005	0	-						
	2002	44.3	-	2005	0	-						
	2003	106.6	-	2004	0	-						
	2003	106.6	-	2004	35.5	-						
	2003	46.8	-	2004	0	-						
	2003	87.9	-	2004	107	-						
	2003	38.7	-	2004	149	-						
	2003	84.1	-	2004	155	-						
	2003	281.4	-	2004	154	-	2005	0	-			
	2003	123.5	-	2004	116	-	2006	147	-			
	2003	56.2	-	2005	0	-						
	2003	348.4	-	2005	0	-						
	2004	370	-	2005	0	-						
	2004	35.5	-	2005	0	-						
	2004	41.9	-	2005	0	-						
	2004	140	-	2005	0	-						
	2004	38.7	-	2005	0	-						
	2004	35.5	-	2005	0	-						
	2004	65	-	2006	107	-						
	2004	107	-	2006	116	-						

Each row corresponds to an individual analyzed several times at intervals of one year or more. Year_1_ corresponds to the first year of analysis and Year_2_, Year_3_, Year_4_ correspond to the following years of re-capture, re-sampling and re-analysis. S, Antibody titer in the serum. C, nRT-PCR results performed on the clot. RT-PCR on blood clots were not done (nd) before 1999. -: negative results by nRT-PCR, +: positive results by nRT-PCR.

### Presence of EBLV-1 RNA in *M. myotis*

Dead bats (n = 17) were collected and analyzed simultaneously by FAT and by nRT-PCR. The FAT results were all negative. In contrast, 2 *M. myotis* collected in refuge 2, in particular the brain of one and the heart and tongue of the other were positive by nRT-PCR.

The blood clots were also analyzed by nRT-PCR and gave 3.68% (n = 136) and 5.17% (n = 290) of positive reactions in colonies 1 and 2, respectively (Table 1). nRT-PCR performed on positive tissues without previous reverse transcription gave negative results. Two of these positive samples (01076, 01077) were further amplified on a larger region of the N gene (Acc. Nb EF577260 and EF577261, respectively), and two of them (01077, 02085) were also amplified by nRT-PCR using primers targeting the glycoprotein gene (Acc. Nb EF577262 and EF577263, respectively). A comparative analysis of all the N 122bp-long sequences amplified (EF187828-EF187847 and EF207412) indicates that the sequences obtained during this study grouped with previously described EBLV-1 [4]. The percentage of divergence was calculated by comparison with 4 different EBLV-1 isolates. It ranges from 0 to 4.9% with a French and a Dutch EBLV-1a isolates (respectively 03002FRA and 9366HOL) and from 1.6 to 4.1 % with two Spanish EBLV-1b isolates (94285SPA and 9483SPA). Conversely this score was higher (from 20.5 to 23%) with a Duvenhage isolate (86132SA) as well as with two EBLV-2 isolates 9018HOL and 9007FIN (from 23 to 27.9%).

Of all the blood samples analyzed by both nRT-PCR and seroneutralization (n = 429), only two were confirmed positive by both techniques (χ ^2^ = 8.91, p<0.01) (Figure 1; Table 1). This result demonstrates the value of this simple sampling technique as a complementary indicator to seroneutralization of EBLV-1 infection in a bat colony.

### Survival of *M. myotis*

The apparent survival rate of colony 1 was calculated from 93 *M. myotis* captured from 2001 to 2006. Among the set of candidate models (Table S2), the best model showed no evidence of sex and time dependence in survival rate (Φ = 0.719, 95% CI, 0.407–0.905). From 2000 to 2006, 799 *M. myotis* were captured in colony 2. The best model (Table S2) also showed a constant survival rate (Φ = 0.708, 95% CI, 0.637–0.769) despite evidence of virus infections, as demonstrated by temporal variations of the seroconversion rate observed in this colony and the RT-PCR results. This observation indicates that the mortality rate was not modified by recurrent epidemic cycles and that the mortality induced by EBLV-1 infection, α, could be considered negligible in our analysis. Survival rates of colonies 1 and 2 were not significantly different.

### Temporal dynamics of EBLV-1 infection

The temporal pattern of EBLV-1 infection was determined by using a dynamic model [14] (Figure S1), the mortality rate (µ = 0.281, 95% CI, 0.095–0.593) and the epidemiological parameters calculated during the longitudinal study (R_0_ = 1.706, and recovery rate, *v* = 0.719, 95% CI, 0.407–0.905). The predicted average duration of infectiousness was D  =  5.077, 95% CI, 4.033 – 8.968.

**Figure 2:**
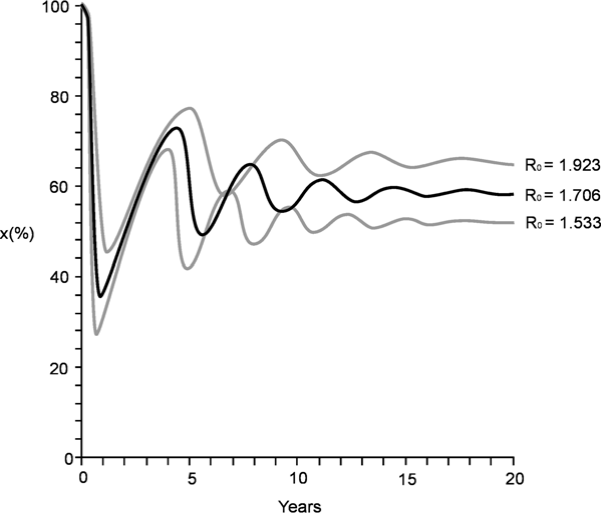
Temporal Pattern Model. Temporal pattern of the number of susceptible bats X(t) in *M. myotis* colonies, obtained by Anderson & May (1991) model, using R_0_ = 1.706 and his 95% confidence interval.

The model shows that after the initial introduction of EBLV-1 in the susceptible bat population, seroprevalence tends to oscillate following periods and amplitudes that diminish year after year until they reach the equilibrium. The results obtained from colony 1 indicate that it is in the final phase of the model and, therefore, the colony is near the equilibrium. However, in colony 2, the oscillations observed in the number of seropositive bats indicate that the colony is in the initial phase of the model (Figure 1 and 2). This analysis proves that there is a good agreement between the predicted and the observed values.

## Discussion

Bats are important reservoir hosts of RNA viruses, including lyssaviruses, which can cross the species barrier to infect humans and other domestic and wild non-flying mammals [1]. In Europe, bat handlers and those entering bat habitats have been provided with guidelines to avoid exposure to lyssaviruses that bats may harbor [22]. These protective measures include rabies vaccinations, protective personal equipment and post-exposure rabies prophylaxis or booster doses of vaccination in case of exposure. However, the policy concerning the maintenance of colonies in buildings and caves open to the public, where rabies-positive bats have been recorded, relies only on advice of experts and is still controversial. To provide epidemiologists and public health officials with data to effectively implement public health measures regarding the conservation of bat populations, we undertook a 12-year field study to identify the temporal dynamics of EBLV-1 in colonies of *M. myotis* bats by combining multidisciplinary approaches. Data collected were used to predict the epidemiological characteristics of EBLV-1 infection in *M. myotis* by means of a simple temporal dynamic model and to estimate the mortality rate.

In nature, the spatial structure of bat populations is variable and may influence the dynamics of infection. Some gregarious bat species have a metapopulation structure consisting of spatial discrete subpopulations and seasonal interaction [1]. As demonstrated by capture-recapture data, the insectivorous bat colonies we studied showed limited exchange and could therefore be considered as spatial discrete subpopulations of a few hundred individuals. To maintain its circulation over the time in such populations, the virus-host relationships should probably follow some constraints in terms of mortality rate, development of immunity and number of susceptible bats.

The significant fluctuations in the percentage of seropositive bats observed in colony 2 are indicative of several different episodes of EBLV-1 infection occurring in *M. myotis* colony during the time of study. These different episodes of EBLV-1 infection did not modify significantly the survival rate of *M. myotis* bats. None of the bats captured (included all the sero- and nRT-PCR-positive) displayed modified behavior that could be related to rabies. Furthermore, 19 bats were shown to survive at least one year after the characterization of EBLV-1-neutralizing antibodies in their serum. These data provide the first evidence that mortality of *M. myotis* in their natural environment does not increase significantly after episodes of EBLV-1 infection. Therefore, our observations support survival in *M*. myotis after EBLV-1 , as suggested for some other bat species maintained in captivity [23]–[25] and more recently for *E. serotinus*, which were found to survive for a long period after EBLV-1 detection in their saliva [8]. Further studies will probably be needed to evaluate more precisely the survival rate of bats after EBLV-1 infection. However, our observation already contrasts with findings in natural conditions in most wild non-flying carnivores [16]–[17] and in other bat species [7], [26]. Therefore, the evaluation of the risk of transmission of lyssavirus to humans cannot be determined on the basis of observations of abnormal mortality in bat colonies.

Rabies infection in non-flying animals is the paradigm for studies of wildlife disease waves [16], [27], [28]. Here we established that EBLV-1 infection in bats follows the same rule. The epidemiological model used [14] is based on human infections and, therefore, it is used as an approach. However, it explains the patterns that follow the lyssavirus infection observed in *Myotis myotis*. Observed and predicted (given by the model) data concerning the variation of percentage of seropositive *M. myotis* are concordant. *Lyssavirus* infection in *M. myotis* is characterized by a high degree of bat immunity after circulation of the virus. This high percentage of seropositive bats after infection is indicative of efficient virus transmission between individuals and rapid circulation of the virus in the colony. These findings are not surprising in a gregarious bat species such as *M. myotis* in which a high contact rate between individuals is facilitated in the roost, where bats are concentrated in less than one square meter. After the initial introduction of EBLV-1 into a susceptible bat population, seroprevalence to this virus tends to oscillate (as observed in colony 2); the amplitude of the oscillations gradually decreases until a steady equilibrium is achieved, as observed in colony 1. These fluctuations in seroprevalence are characteristic of infections that induce long-lasting immunity [14], [29]. Bats recover from infection and develop a degree of immunological protection to future infection. Recapture data and individual serological follow-up show that this immunity can persist for more than one year. The delay between the waves is then dependent on the rate of inflow of susceptible bats into the colonies as a consequence of new births, immigration of naïve animals from neighboring colonies, and expiration of EBLV-1-specific immunity in previously infected animals. When a sufficient fraction of susceptible individuals in the bat population is reached, the virus spreads again if infected individuals join the colony.

Having determined that the model corroborates observed features of the dynamics of EBLV-1 infection in *M. myotis* bats, we used it as a basis to further study the local spread of infection. This allowed us to predict, for the first time, the R_0_ of *Lyssavirus* in an insectivorous bat colony. The R_0 _value obtained (R_0_ = 1.706) was lower than those recorded for most human infectious diseases [14]. The prediction of the average duration of infectiousness (D = 5.1 days) is also new. This predicted value is comparable to those observed in experimental conditions in *Eptesicus fuscus* [30] and in natural conditions in raccoons [31] and dogs [32]–[34] but shorter than that recorded in foxes [35].

In summary, this study demonstrates cyclic EBLV-1 infection in spatial discrete subpopulations of *M. myotis* in Spain with periodic oscillations in the number of susceptible, immune and infected bats without any significant increase in associated mortality. Immunity persists in some of the bats for more than one year. The temporal dynamic model predicted a low basic reproductive rate and a short infectious period. These observations could be attributed to a long establishment of the infection in this species and virus-host co-evolution, as already indicated by the low rate of evolutionary change in EBLV-1 and by the low viral growth rate in the bat population [5]. These findings are highly significant for the development of measures to protect humans from the risk of lyssavirus infection from bats. It confirms the potential risk of rabies transmission to humans. This study also highlights the importance of the dynamics of infection due to local spread within the colony, as compared to long-range spread. However, recurrent epidemics are probably triggered by the exchange of infective bats between colonies. Greater understanding of the spatio-temporal dynamics of *Lyssavirus* infection in bats also provides useful information for epidemiologists and public health officials to forecast the spread and severity of future outbreaks of (re)emerging viruses circulating in bats.

## Material and Methods

### Capture of bats and collection of specimens and data

Biological samples and information about the seasonal movements of bats and virus circulation were obtained from two bat colonies in the locations of Inca and Llucmajor on Mallorca (Balearic Islands, Spain), in which EBLV-1 infection was previously demonstrated [18]. All captured bats were sexed and banded on the forearm to facilitate the monitoring of individual infection and their movements between colonies. Bats were captured in colony 1 from 1995 to 2006 during the daytime by means of a net. In the last year very few blood samples were collected and they have not been considered. During the same period bats were captured in colony 2 at night, when they left for foraging, by means of a net that entirely covered the entrance of the cave. In both cases the capture method was always carried out in the same way throughout the study. The few bat carcasses analyzed during the study correspond to bats found dead during the fieldworks and to the very few which unfortunately died during handling. They were stored at −80°C before analysis. Blood samples were taken from all captured bats from colony 1 [18]. As for colony 2, the bats captured during the night were kept into cotton bags. Further, some of them were randomly sampled for blood analyses.

The sizes of the both colonies were calculated indirectly by the capture-recapture technique. The trap sessions were carried out in spring over six- (2001–2006 for colony 1) and seven- (2000–2006 for colony 2) year periods respectively. The sizes of the colonies were estimated using the Jolly-Seber methodology for open population incorporated in the Mark program [36].

### Laboratory investigations

Brain, pharynx-esophagus, larynx, lung, heart and tongue samples were collected from dead bats [26]. Fluorescent antibody test (FAT) was performed on brain tissue specimens using polyclonal fluorescent isothiocyanate-labelled rabbit anti-rabies nucleocapsid immunoglobulin G (Bio-Rad) [26]. Serum and blood clot collected from captured bats were analyzed separately. Total RNA was extracted from blood clots and organs and tested by nRT-PCR using specific primers (N60-N41, N62-N63) targeting the EBLV-1 nucleoprotein gene [3], [18]. Positive PCR products were further sequenced and compared, as previously described [3], [5]. As a confirmation method, some of the positive RNA samples were further tested by nRT-PCR using primers N127-N499 (5′-CTGCCACATTGGTCTTATAG-3′, position 479 to 499 of the N coding region of the rabies virus genome), N60-N41, targeting a larger region of the nucleoprotein gene [5]. Two positive RNA samples were also amplified by nRT-PCR using primers G594 (5′-TCCAGAGAATCCTAAACCCG-3′, position 594 to 604 of the G coding region of the rabies genome)-G1197 (5′-GCTCAATGTGCTGCTGTAAC-3′, position 1197 to 1207 of the G coding region of the rabies genome), G641 (5′-GCAAAGGAAAGAAAGCAACC-3′, position 604 to 641 of the G coding region of the rabies genome)-G1165 (position 1165 to 1185 of the G coding region of the rabies genome). The technique used for the detection of EBLV-1 antibodies is an adaptation of the Rapid Fluorescent Focus Inhibition Test (RFFIT) [18], [37]. A constant dose of a previously titrated, cell culture adapted EBLV-1 challenge virus 8918FRA was incubated with 3 fold dilutions of the sera to be titrated. After incubation of the serum/virus mixtures, a suspension of BSR cells was added. After 24 hours incubation, the cell monolayer was acetone-fixed and stained with a fluorescent anti-nucleocapsid antibody (BioRad, Marnes-la-Coquette, France) to detect the presence of non-neutralized virus (fluorescent foci). Titers are presented as an arithmetic mean of two independent repetitions. Serum samples with antibody titers <27 are considered negative for EBLV-1 neutralizing antibodies.

The annual percentages of seroprevalence were compared by a Chi-square test.

### Analysis of the sequences

A comparative analysis was performed on a 122 bp sequence of the lyssavirus nucleoprotein gene using clustalx 1.83.3 [38]. Sequences of isolates (03002FRA, Acc. Nb. AY863381; 9366HOL, Acc. Nb. AY863359; 94285ESP, Acc. Nb. AY963391; 9483ESP, Acc. Nb. AY863390; 9018HOL, Acc. Nb. AY863403; 9007FIN, Acc. Nb. AY863406) representative of EBLV-1 and EBLV-2 diversity [5] were selected for comparison. Duvenhage isolate 86132SA (Acc. Nb. AY996323) was also used for comparison.

We partially sequenced the gene of cytochrome b to confirm that the captured animals belonged to *M. myotis* [39].

### Survival rate

The apparent survival probabilities (Φ) of *M. myotis* from colonies 1 and 2 were estimated over a six- (2001–2006) and seven- (2000–2006) year period, respectively. Years previous to 2000 were excluded from analysis since the capture-recapture method was performed in a different way. The juvenile individuals were excluded from analysis. Survival rate was modeled following the capture-mark-recapture methodology [40], using the Mark program [41]. Model selection method was used to find the most parsimonious model from a set of candidate models [40] (Table S2).

### Temporal dynamics of EBLV-1 infection

Basic Reproductive Rate (R_0_) of the virus in *M. myotis* was calculated using the equation R_0_ = 1/x*, where x* represents the host population fraction that is susceptible at equilibrium [14]. The value of x* was estimated from serological data observed in colony 1 from 1995 to 2005. The recovery rate (*v*) was calculated for colony 1 by equations Nos. 6.4, 6.11, 6.12 [14]. The average duration of infectiousness (D) was obtained using the equation D = 1/*v*.

A dynamic model with 3 categories was used to predict the temporal pattern of EBLV-1 infection (Figure S1). The bat population was divided into susceptible (X), infected (Y) and immune (Z). Changes of percentages in categories were obtained by feeding the model with the ecological and epidemiological data collected and in particular the differential equations Nos. 6.1–6.4, as proposed by Anderson and May [14]. The model was evaluated using Maple v9.01 software.

## Supporting Information

Table S1 No. of Recaptured and Analyzed *M. myotis* in Colonies 1 and 2, 1996–2006. ^a^Successive analyses in the same individuals were made at intervals of ≥1 year
(0.03 MB DOC)

Table S2 Modeling Survival Probabilities of M. myotis in Colonies 1 and 2.
(0.07 MB DOC)

Figure S1 Diagram of the Flow of Hosts between Susceptible. This diagram shows the dynamics of the interaction between a directly transmitted virus and its host population. The host dies at a per capita rate γ. The infected host experiments an additional death rate α, induced by virus infection and a seroconversion rate υ. The transmission coefficient β determines the rate at which new infection arises as a consequence of mixing between susceptible and infected individuals.
(0.49 MB TIF)

## References

[1] Calisher CH, Childs JE, Field HE, Holmes KV, Schountz T (2006). Bats: important reservoir hosts of emerging viruses.. Clin Microbiol Rev.

[2] Rupprecht CE, Hanlon CA, Hemachudha T (2002). Rabies re-examined.. Lancet Infect Dis.

[3] Amengual B, Whitby JE, King A, Serra-Cobo J, Bourhy H (1997). Evolution of European bat lyssaviruses.. J Gen Virol.

[4] Brookes SM, Aegerter JN, Smith GC, Healy DM, Jolliffe TA (2005). European Bat Lyssavirus in Scottish Bats.. Emerg Infect Dis.

[5] Davis P, Holmes E, Larrous F, Van der Poel WH, Tjornehoj K (2005). Phylogeography, Population Dynamics, and Molecular Evolution of European Bat Lyssaviruses.. J Virol.

[6] Echevarría JE, Avellón A, Juste J, Vera M, Ibáñez C (2001). Screening of Active Lyssavirus Infection in Wild Bat Populations by Viral RNA Detection on Oropharyngeal Swabs.. J Clin Microbiol.

[7] Van der Poel WHM, van der Heide R, Verstraten ERAM, Takumi K, Lina PHC (2005). European bat lyssaviruses, the Netherlands.. Emerg Infect Dis.

[8] Vázquez S, Ibáñez C, Juste J Echevarría JE, Dodet B, Schudel A, Pastoret PP, Lombard M (2006). EBLV1 Circulation in Natural Bat Colonies of *Eptesicus serotinus*: a Six Year Survey.. Rabies in Europe. Dev Biol.

[9] Davis P, Bourhy H, Holmes EC (2006). The Evolutionary History and Dynamics of Bat Rabies Virus.. Infect Genet Evol.

[10] Anonymous (2003). Summary of rabies cases in Europe. Rabies Bulletin Europe 27(1):1.. http://www.who-rabiesbulletin.org/Journal/Archive/Bulletin_2003_1.PDF.

[11] Fooks AR, McElhinney LM, Pounder DJ, Finnegan CJ, Mansfield K (2003). Case report: Isolation of a European bat lyssavirus type 2a from a fatal human case of rabies encephalitis.. J Med Virol.

[12] Muller T, Cox J, Peter W, Schafer R, Johnson N (2004). Spill-over of European Bat Lyssavirus Type 1 into a Stone Marten (*Martes foina*) in Germany.. J Vet Med.

[13] Ronsholt L (2002). A New Case of European Bat Lyssavirus (EBL) Infection in Danish Sheep.. Rabies Bull Europe.

[14] Anderson RM, May RM (1991). Infectious Diseases of Humans..

[15] Childs JE, Curns AT, Dey ME, Real LA, Feinstein L (2000). Predicting the local dynamics of epizootic rabies among raccoons in the United States.. Proc Natl Acad Sci U S A.

[16] Coyne MJ, Smith G, McAllister FE (1989). Mathematic model for the population biology of rabies in raccoons in the mid-Atlantic states.. Am J Vet Res.

[17] Harris S, White PCL, King A, Fooks AR, Aubert M, Wandeler AI (2004). Epidemiological Models.. Rabies in Europe and the Mediterranean Basin.

[18] Serra-Cobo J, Amengual B, Abellán C, Bourhy H (2002). European Bat *Lyssavirus* Infection in Spanish Bat Populations.. Emerg Infect Dis.

[19] Harris SL, Brookes SM, Jones G, Huston AM, Racey PA, Aegerter J (2006). European bat lyssaviruses: distribution, prevalence and implications for conservation.. Biological Conservation.

[20] Lina PH, Hutson AM (2006). Bat rabies in Europe: a review.. Dev Biol (Basel).

[21] Mitchell-Jones AJ, Amori G, Bogdanowicz W, Krystufek B, Reijnders PJH (1999). The Atlas of European Mammals..

[22] Bourhy H, Dacheux L, Strady C, Mailles A (2005). Rabies in Europe in 2005.. Euro Surveill.

[23] Aguilar-Setien A, Loza-Rubio E, Salas-Rojas M, Brisseau N, Cliquet F (2005). Salivary excretion of rabies virus by healthy vampire bats.. Epidemiol Infect.

[24] Baer GM, Bales GL (1967). Experimental rabies infection in the Mexican freetail bat.. J Infect Dis.

[25] Wellenberg GJ, Audry L, Ronsholt L, van der Poel WH, Bruschke CJ (2002). Presence of European bat lyssavirus RNAs in apparently healthy *Rousettus aegyptiacus* bats.. Arch Virol.

[26] Bourhy H, Kissi B, Lafon M, Sacramento D, Tordo N (1992). Antigenic and molecular characterization of bat rabies virus in Europe.. J Clin Microbiol.

[27] Bacon PJ, Bacon PJ (1985). A System Analysis of Wildlife Rabies Epizootics.. Population Dynamics of rabies in wildlife.

[28] Grenfell B (2002). Rivers dam waves of rabies.. Proc Natl Acad Sci U S A.

[29] Cox FEG (1993). Modern parasitology..

[30] Shankar V, Bower RA, Davis AD, Rupprecht CE, O'Shea TJ (2004). Rabies in captive colony of big brown bats (*Eptesicus fuscus*).. J Wild Dis.

[31] Bigler WJ, McLean RG, Trevino HA (1973). Epizootiologic aspects of raccoon rabies in Florida.. Am J Epidemiol.

[32] Fekadu M (1988). Pathogenesis of rabies virus infection in dogs.. Rev Infect Dis.

[33] Tepsumethanon V, Lumlertdacha C, Sitprija V, Meslin FX, Wilde H (2004). Survival of naturally infected rabid dogs and cats.. Clin Infect Dis.

[34] Vaughn JB, Gerhardt P, Newell KW (1965). Excretion of street rabies virus in saliva of dogs.. J Am Med Assoc.

[35] Aubert MF, Blancou J, Barrat J, Artois M, Barrat MJ (1991). Transmissibility and pathogenicity in the red fox of two rabies viruses isolated at a 10 year interval.. Ann Rech Vet.

[36] Pollock RH, Nichols JA, Brownie C, Hines JE (1990). Statistical inference for capture-recapture experiments.. Wildl Monogr.

[37] Reynes JM, Molia S, Audry L, Hout S, Ngin S (2004). Serologic evidence of lyssavirus infection in bats, Cambodia.. Emerg Infect Dis.

[38] Thompson JD, Gibson TJ, Plewniak F, Jeanmougin F, Higgins DG (1997). The ClustalX windows interface: flexible strategies for multiple sequence alignment aided by quality analysis tools.. Nucleic Acids Res.

[39] Bickham JW, Patton JC, Schlitter DA, Rautenbach IL, Honeycutt RL (2004). Molecular phylogenetics, karyotypic diversity, and partition of genus *Myotis* (Chiroptera: Vespertilionidae).. Mol Phylogenet Evol.

[40] Lebreton JD, Burnham KP, Clobert J, Anderson DR (1992). Modelling survival and testing biological hypotheses using marked animals: a unified approach with case studies.. Ecol Monogr.

[41] White GC, Burnham KP (1999). Program MARK: survival estimation from populations of marked animals.. Bird Study.

