# Supplementary material for: Temporal Dynamics of European Bat Lyssavirus Type 1 and Survival of Myotis myotis Bats in Natural Colonies
Source: PLoS One. 2007 Jun 27;2(6):e566. doi: 10.1371/journal.pone.0000566 (PMC1892799; doi:10.1371/journal.pone.0000566)
Supplement: Table S1 — No. of Recaptured and Analyzed M. myotis in Colonies 1 and 2, 1996–2006. aSuccessive analyses in the same individuals were made at intervals of ≥1 year (0.03 MB DOC) [file pone.0000566.s001.doc]

Table S1. No. of Recaptured and Analyzed *M. myotis* in Colonies 1 and 2, 1996-2006.

| **Category** | **Number** |
| --- | --- |
|  |  |
| No. of bats banded in the colonies 1 and 2 | 1260 |
| No. of bats banded and recaptured in the same colony | 352 |
| No. of bats banded that moved between colonies 1 and 2 | 3 |
| No. of bats banded and analyzed^a^ |  |
| Once | 406 |
| Twice | 72 |
| Three times | 12 |
| Four times | 3 |
| No. of bats analyzed and not banded | 45 |

aSuccessive analyses in the same individuals were made at intervals of ≥ 1 year.
