# Supplementary material for: Temporal Dynamics of European Bat Lyssavirus Type 1 and Survival of Myotis myotis Bats in Natural Colonies
Source: PLoS One. 2007 Jun 27;2(6):e566. doi: 10.1371/journal.pone.0000566 (PMC1892799; doi:10.1371/journal.pone.0000566)
Supplement: Table S2 — Modeling Survival Probabilities of M. myotis in Colonies 1 and 2. (0.07 MB DOC) [file pone.0000566.s002.doc]

Table S2. Modeling Survival Probabilities of *M. myotis* in Colonies 1 and 2.

| **Colony 1** | |  |  |  |  |  |
| --- | --- | --- | --- | --- | --- | --- |
| **No.** | **Model name** | **QAIC_c_** | **ΔQAIC_c_** | **W*_i_*** | **K** | **Deviance** |
| **1** | **, *p*** | **106.17** | **0.00** | **0.50** | **2** | **32.87** |
| 2 | φ_s_ , *p* | 108.14 | 1.97 | 0.19 | 3 | 32.72 |
| 3 | φ_t_ , *p* | 114.40 | 8.22 | 0.08 | 6 | 32.38 |
| 4 | φ_s *t_ , *p* | 125.10 | 18.92 | 0.00 | 11 | 31.19 |
| 5 | φ_s *t_ , *p*_s_ | 127.47 | 21.29 | 0.00 | 12 | 31.03 |
| 6 | φ_s *t_ , *p* _t_ | 132.07 | 25.89 | 0.00 | 14 | 30.41 |
| 7 | φ_s *t_ , *p*_s *t_ | 137.47 | 31.30 | 0.00 | 18 | 24.68 |
|  | |  |  |  |  |  |
| **Colony 2** | |  |  |  |  |  |
| **No.** | **Model name** | **QAIC_c_** | **ΔQAIC_c_** | **W*_i_*** | **K** | **Deviance** |
| **1** | **, *p*(f)_t_ *p*(m)** | **1297.32** | **0.00** | **0.78** | **8** | **88.48** |
| 2 | , *p*_s *t_ | 1300.98 | 3.66 | 0.12 | 13 | 81.92 |
| 3 | φ_s_ , *p*_s *t_ | 1303.03 | 5.71 | 0.04 | 14 | 81.92 |
| 4 | φ_t_ , *p*_s *t_ | 1305,15 | 7.83 | 0.01 | 17 | 77.85 |
| 5 | φ_s *t_ , *p* _t_ | 1312.60 | 15.28 | 0.00 | 17 | 85.30 |
| 6 | φ_s *t_ , *p*_s *t_ | 1312.81 | 15.49 | 0.00 | 22 | 75.09 |
| 7 | φ_s *t_ , *p*_s_ | 1332.79 | 35.47 | 0.00 | 14 | 111.68 |
| 8 | φ_s *t_ , *p* | 1333.06 | 35.74 | 0.00 | 13 | 114.01 |

Modelling survival (φ) and capture probabilities (*p*) of *M. myotis*, as function of time (t) and sex (s), starting from model φ_s *t_ , *p*_s *t_; QAIC_c_= Akaike’s information criterion, quasi-likelihood corrected; ΔQAIC_c_= QAIC_c_-based diference to the best model; W*_i_* = normalized Akaike weight of model i; K = number of estimated parameters; f = females; m = males. The best supported model is in bold.
