# Supplementary figures and images for: Temporal Dynamics of European Bat Lyssavirus Type 1 and Survival of Myotis myotis Bats in Natural Colonies
Source: PLoS One. 2007 Jun 27;2(6):e566. doi: 10.1371/journal.pone.0000566 (PMC1892799; doi:10.1371/journal.pone.0000566)

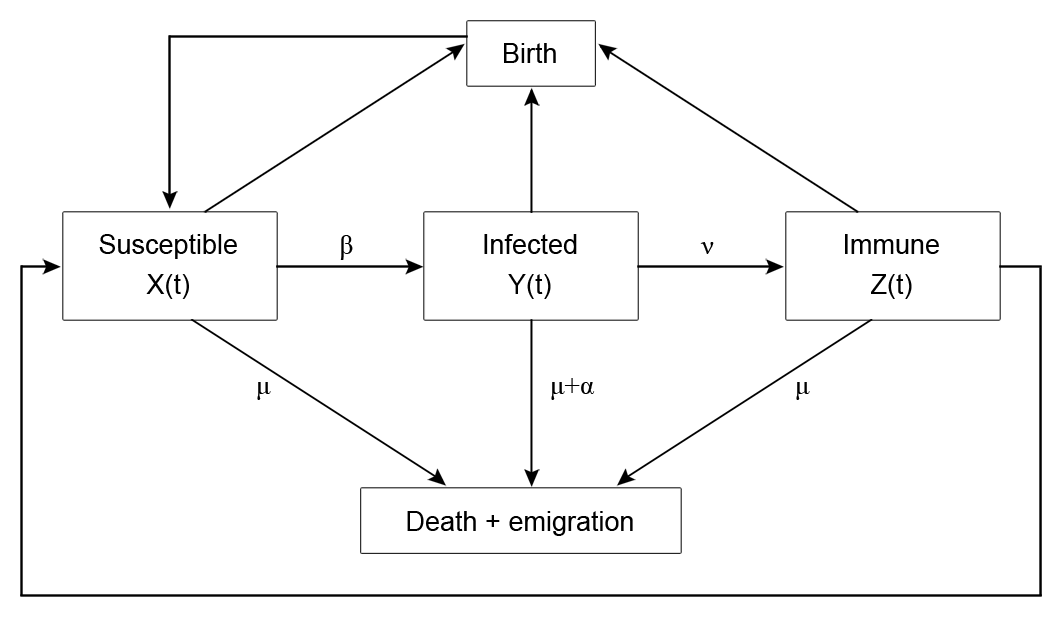

Supplement: Figure S1 — Diagram of the Flow of Hosts between Susceptible. This diagram shows the dynamics of the interaction between a directly transmitted virus and its host population. The host dies at a per capita rate γ. The infected host experiments an additional death rate α, induced by virus infection and a seroconversion rate υ. The transmission coefficient β determines the rate at which new infection arises as a consequence of mixing between susceptible and infected individuals. (0.49 MB TIF) [file pone.0000566.s003.tif]
